# PREVENTION OF SEVERE EVENTS IN OLDER PEOPLE IN SWEDEN – POSSIBILITIES AND CHALLENGES

**DOI:** 10.1093/geroni/igae098.4053

**Published:** 2024-12-31

**Authors:** Marie Ernsth Bravell, Linda Johansson, Deborah Finkel

**Affiliations:** Jonkoping University, Jönköping, Jonkopings Lan, Sweden; School of Health and Welfare, Jönköping University, Jönköping, Jonkopings Lan, Sweden; Jönköping University, Jönköping University, Jonkopings Lan, Sweden

## Abstract

Older adults are at high risk of malnutrition, falls, pressure ulcers and poor oral health. In Sweden the quality register Senior Alert is trying to improve the preventive work among older people. This study used data from Senior Alert to understand how the preventive work process functions in different care settings. Data included 2957 persons that were assessed in Senior Alert: 864 in nursing homes, 226 in home care and 1829 in hospital. Descriptive and comparative analyses were performed to understand how many were assessed as having risk, and then the actions planned and follow-ups. The results demonstrated that people who were assessed in hospital had risks of some kind in almost half of the cases (46%), in comparison to nursing homes (29%) and home care (8%). Planned actions differed between the settings: in nursing homes, actions were more often planned to prevent poor oral health (29%) whereas hospital staff more often planned actions to prevent falls (73%), malnutrition (71%) and pressure ulcers (57%). Planned actions were significantly less in home care. Follow-ups follow the same patterns, where follow-up in home care were significantly smaller than in nursing homes and hospitals in all areas. Home care provides good opportunities to provide preventive care, as it is more continuous than, for example, hospital care. Additional analyses identified particular actions planned and performed, for a fine-grained investigation of preventive care in Sweden.

